# A nanoliposome delivery system to synergistically trigger TLR4 AND TLR7

**DOI:** 10.1186/1477-3155-12-17

**Published:** 2014-04-26

**Authors:** Christopher B Fox, Sandra J Sivananthan, Malcolm S Duthie, Julie Vergara, Jeffrey A Guderian, Elliot Moon, David Coblentz, Steven G Reed, Darrick Carter

**Affiliations:** 1 Infectious Disease Research Institute (IDRI), Seattle, WA, USA; 2 DFNet Research, Seattle, WA, USA; 3 PAI Life Sciences, 1616 Eastlake Ave E Suite 500, Seattle 98102 WA, USA

**Keywords:** TLR4, TLR7, Synergy, Adjuvant, Manufacturability, Liposome

## Abstract

**Background:**

Recent reports that TLR4 and TLR7 ligands can synergistically trigger Th1 biased immune responses suggest that an adjuvant that contains both ligands would be an excellent candidate for co-administration with vaccine antigens for which heavily Th1 biased responses are desired. Ligands of each of these TLRs generally have disparate biochemical properties, however, and straightforward co-formulation may represent an obstacle.

**Results:**

We show here that the TLR7 ligand, imiquimod, and the TLR4 ligand, GLA, synergistically trigger responses in human whole blood. We combined these ligands in an anionic liposomal formulation where the TLR7 ligand is in the interior of the liposome and the TLR4 ligand intercalates into the lipid bilayer. The new liposomal formulations are stable for at least a year and have an attractive average particle size of around 140 nm allowing sterile filtration. The synergistic adjuvant biases away from Th2 responses, as seen by significantly reduced IL-5 and enhanced interferon gamma production upon antigen-specific stimulation of cells from immunized mice, than any of the liposomal formulations with only one TLR agonist. Qualitative alterations in antibody responses in mice demonstrate that the adjuvant enhances Th1 adaptive immune responses above any adjuvant containing only a single TLR ligand as well.

**Conclusion:**

We now have a manufacturable, synergistic TLR4/TLR7 adjuvant that is made with excipients and agonists that are pharmaceutically acceptable and will have a straightforward path into human clinical trials.

## Background

Modern, effective vaccines rely on a combination of a purified antigen against which an immune response is desired and an adjuvant that triggers the innate immune system to enhance the magnitude and quality of the generated immune response [1]. Producing new adjuvants that can direct appropriate immunity is therefore becoming key to vaccine design and groups have shown that even in the context of the same antigen different outcomes are achieved as a function of the co-administered adjuvant [2]. Recent commercial approval of the Cervarix® vaccine that contains MPL, a defined TLR4 ligand, has added momentum to the development of a new generation of adjuvants [3].

The immune system has the inborn ability to recognize molecular signatures carried by microbes. These Microbe Associated Molecular Patterns (MAMPs) trigger any of an array of receptor sensors in cells to alert the organism and mobilize an appropriate immune response. MAMPs are generally molecules that are vital to the microbe, performing key functions, but are not found in the host. Examples include: lipopolysaccharides (LPS) found on the surface of gram negative bacteria [4], RNAs produced as part of viral replication [5], and flagellins that make up bacterial flagella [6].

The Toll-Like Receptor (TLR) family of proteins is a well characterized group of innate signaling receptors that respond to a variety of MAMPs. TLRs 1,2, and 6 hetero-dimerize and respond to lipopeptides; TLR3 binds double stranded RNAs; TLR4 signals when triggered by LPSs; TLR5 senses flagellins; TLR7 and 8 detect single stranded RNAs and TLR9 responds to DNA with CpG motifs [5,7-12]. To augment their ability to appropriately detect pathogens, the TLRs are partitioned where the likely source of the MAMP would be found; i.e. shed LPS is picked up by lipopolysaccharide binding protein and transported to the cell membrane where TLR4 is located and the viral RNA sensors TLR3, 7, and 8 are located in endosomes inside the cell where viral RNAs would be produced and their detection required [13,14].

TLR signaling and the resulting innate and adaptive responses can be enhanced by synergy within the TLR family [15] or by signals from TLR combined with those from non-TLR innate sensors [16,17]. Within the TLR family, TLR4 and TLR7 have been targeted by agonists that are in late stage development [18,19] or are commercial [20,21] and the two TLR show potential for powerful synergism. This can be seen in increased magnitudes of cytokine secretion, enhanced germinal center formation, class switching and antibody diversity [22,23]. While the synergy that is induced is dramatic, the agonists have to be co-localized and efforts to develop such a combination adjuvant that can be manufactured cost effectively at scale are still needed. Researchers in academic and company settings have started to produce adjuvants and vaccine nanoparticles that contain multiple innate ligands [22,24-26] and standard vaccine preparations usually contain several TLR ligands since they are derived from inactivated pathogens [27,28], but most of these attempts are proof of concept exercises that have not progressed due to the cost of manufacturing or the poor definition of the relative ligand content. We report here the development of a nanoliposome that co-localizes TLR4 and TLR7 agonists and synergistically enhances immune responses. The process used for manufacture of the combination can be scalable and commercially viable like similar liposomal formulations.

## Results

### In vitro synergy of GLA and IMQ

**Figure 1:**
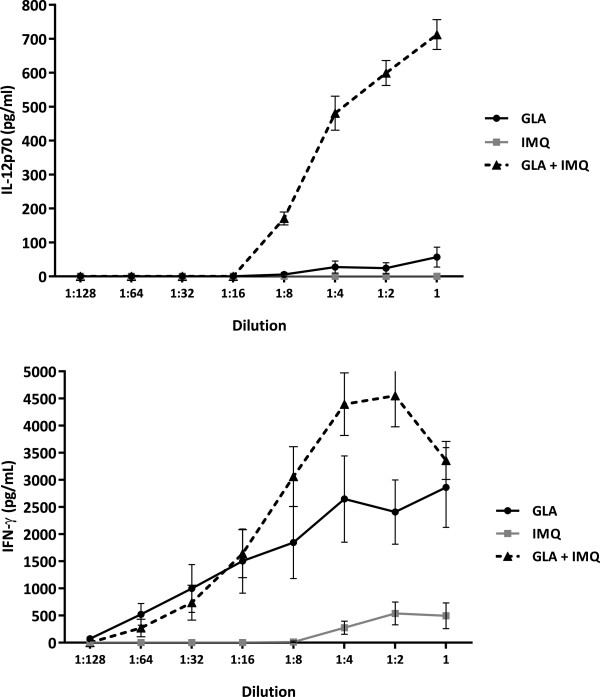
**Synergy between GLA and IMQ *in vitro*.** Human whole blood was incubated with 2 μg/mL of GLA and 8 μg/mL of IMQ either alone or in combination; the amount of secreted IL12p70 (top panel) or IFNγ (bottom panel) were then determined by ELISA. Means and standard error of three donor values are shown. Similar trends were seen for MIP1β and IL8 as well (data not presented).

To demonstrate that the selected ligands would synergize, admixtures of the TLR7 agonist IMQ and the TLR4 agonist GLA were suspended in an aqueous formulation and various dilutions tested for the ability to elicit cytokine secretion from human whole blood. Although IMQ itself is essentially insoluble in water, a proprietary composition by Invivogen facilitates aqueous suspension [29] and, because both agonists act on the same cells, there is no need for co-delivery *in vitro*. As shown in Figure 1, the molecules synergistically elicit secretion of IL12p70 and IFNγ from cells. IL8 and MIP-1β were also determined and demonstrated enhanced secretion in the combination group compared to the single ligands alone. These data demonstrate that when the same cells are stimulated by both ligands, synergistic signaling by the innate immune system can occur.

### Manufacturing of the synergistic nanoliposome

After establishing that the molecules have the potential for synergistic signaling, we set out to develop a manufacturing process that would allow them to be co-localized in the same particle. Liposomes comprise a versatile formulation platform since they can be used to bring together active molecules with different structural properties. For instance, lipidic molecules such as MPL® or GLA can be incorporated in the phospholipid bilayer whereas small, more soluble molecules may be encapsulated in the aqueous core. Furthermore, several liposome-based formulations have been approved for human use or reached advanced clinical development in existing vaccine (e.g. Epaxal®, Mosquirix) and drug (e.g. Ambisome®) products. Finally, liposomes can be manufactured at small diameter (<200 nm), permitting terminal sterile filtration and improved lymph node targeting [30].

The formulation of IMQ presents a particular challenge due to its insolubility in aqueous solutions at physiological pH [31,32]. In fact, IMQ has very low solubility in most organic solvents, with the exception of fatty acids which form the basis of the approved topical cream containing IMQ called Aldara® [31]. IMQ is soluble in acidic aqueous solutions and 0.1 M lactic acid-based formulations of IMQ are in clinical development for the treatment of bladder cancer [32-34]. Although lactic acid is a widely used pharmaceutical excipient [32,35], it does not prevent the systemic distribution of IMQ; therefore, improving its vaccine adjuvant properties requires further formulation techniques to localize the molecule in the body after administration [32,36]. We hypothesized that liposomes with a lactic acid core would facilitate encapsulation of soluble IMQ while enabling the bulk aqueous phase external to the liposomes to maintain a physiological buffer at close to a neutral pH, which is desirable to minimize injection pain [37]. Moreover, liposomes should allow the intercalation of the six acyl chains of GLA into the lipid bilayer.

Liposomes were manufactured using a thin-film method wherein phospholipids and GLA were mixed with cholesterol in organic solvent followed by evaporation of the solvent under vacuum. Liposomes were rehydrated in 100 mM lactic acid containing 10 mg/ml IMQ, and sonicated in a water bath at ~60°C (above the phase transition temperatures of the lipids) for ~1.5 – 3 hrs. The liposomes were then transferred to a desalting column and the external lactic acid solution was exchanged for phosphate-buffered saline (PBS) at pH 7.2.

Different phospholipid compositions previously developed in our lab for formulation of GLA [38] were evaluated as an initial formulation screen (Table 1), including an anionic liposome (DPPC, DPPG, cholesterol; 18:2:5 weight ratio), a cationic liposome (DPPC, DPTAP, cholesterol; 18:2:5 weight ratio), and a neutral liposome (DOPC, cholesterol; 20:5 weight ratio). Total DPPC or DOPC concentrations were estimated at 50 and 55 mg/ml, respectively, assuming no extra loss in the buffer exchange and filtration steps. The cationic liposomes showed lower encapsulation efficiency. Precipitation was evident in the neutral DOPC-based liposomes and substantial IMQ was lost upon filtration through a 0.2-μm membrane, even though average particle size was small and encapsulation efficiency was high. The anionic liposomes also showed comparatively high encapsulation efficiency and much less tendency for precipitation over time. The loss in IMQ concentration upon filtration may represent the proportion of liposomes that are larger than 200 nm and not able to pass through the membrane. Particle size and IMQ concentration in three additional anionic liposome batches prepared at a concentration of 38 mg/ml DPPC and stored at 5°C demonstrated only 7 ± 5% and 9 ± 1% change in particle size and IMQ concentration, respectively, at the 12-month timepoint compared to immediately after manufacture. Finally, one of these three batches also contained GLA, and its concentration changed only ~8% over 12 months. Therefore, the anionic liposomes consisting of DPPC, DPPG, and cholesterol were selected as the most suitable formulation for a synergistic adjuvant containing GLA and IMQ.

**Table 1:** Liposome formulation screen for encapsulation of IMQ

**Liposome type**	**Composition**	**Number of batches**	**IMQ concentration (mg/ml)**	**IMQ loading efficiency (%)**	**% Loss after 0.2-μm filtration**	**Particle size (Zave, nm)**	**Polydispersity index**
Anionic	DPPC/DPPG/Cholesterol*	8	0.6 ± 0.1	6 ± 1	23 ± 12	151 ± 20	0.17 ± 0.07
Cationic	DPPC/DPTAP/Cholesterol	1	0	0	NM	64	0.07
Neutral	DOPC/Cholesterol*	4	0.7 ± 0.2	7 ± 2	45 ± 22	73 ± 23	0.20 ± 0.01

*Some batches also contained GLA, although the presence of GLA did not have a significant effect on the reported IMQ loading efficiency or the other properties mentioned. NM: not measured.

### Biophysical characterization of the liposome

Due to the insolubility of IMQ in aqueous solutions near neutral pH, it was thought that the IMQ should be localized in the acidic interior of the liposomes. We first sought to confirm that the external lactic acid was removed by the desalting column treatment by measuring pH values before and after exposure to the desalting column in liposomes containing IMQ, GLA, both of the agonists, or neither. The pH values prior to buffer exchange ranged from 2.5 - 3.5, whereas pH values after buffer exchange were 6.7 - 6.9 (Table 2), indicating that the lactic acid solution had indeed been replaced with the buffered saline, although the pH values had gradually decreased by an average of ~0.4 units when measured one month after manufacture. After three successive ultracentrifugation and wash steps, 74 ± 5% of IMQ was recovered in the liposome pellet, whereas in an IMQ control solution (not containing liposomes) the IMQ remained in the supernatant. That more IMQ was not recovered in the pellet most likely represents a limitation of the assay since negligible IMQ was present in the supernatant. Together, these results indicate that the majority of IMQ is localized in the lactic acid core of the liposomes. An unexpected result of IMQ incorporation in liposomes was the significantly higher particle size compared to liposomes containing GLA or liposomes without TLR agonists (Table 2), indicating that there may be some physicochemical interaction between the lipids and IMQ. The gradual decrease in pH over time is an important point that may be addressable through further liposome optimization. However, even if buffer exchange caused the internal pH of the liposomes to change enough to affect imiquimod solubility, the result could be the same type of ‘coffee-bean’ appearance that has been demonstrated for the FDA-approved liposome formulation of the cancer drug doxorubicin known as Doxil [39].

**Table 2:** Comparison of liposome properties

**Liposome type**	**Number of batches**	**Particle size (Z-ave, nm)**	**Polydispersity index**	**GLA retention efficiency (%)**	**pH before buffer exchange**	**pH after buffer exchange**
GLA-LS	4	64 ± 12	0.23 ± 0.05	81 ± 5	2.54 ± 0.06	6.73 ± 0.10
IMQ-LS*	4	160 ± 25	0.21 ± 0.10	-	3.48 ± 0.01	6.84 ± 0.10
GLA/IMQ-LS*	4	141 ± 5	0.14 ± 0.02	79 ± 10	3.53 ± 0.01	6.92 ± 0.01
LS	4	70 ± 25	0.22 ± 0.04	-	2.60 ± 0.05	6.81 ± 0.07

*These liposomes are part of the 8 total IMQ-containing liposomes represented in row 1 of Table 1.

While IMQ loading efficiency is low (Table 1), it should be noted that the loading mechanism is passive rather than the more efficient, active loading methods based on pH or ammonium sulfate gradients [40], although some aqueous solubility is a prerequisite of such loading methods. By employing the mathematical model developed by Xu et al. [41], assuming 75 mM phospholipid concentration, a monodisperse average particle size of 160 nm with polydispersity width of ± 35.2 nm, a bilayer thickness of 4.8 nm, and an average lipid molecular area of 0.4 nm^2^[41,42], the expected passive encapsulation efficiency of IMQ is predicted to be ~24%. This may be overly optimistic considering that the Z-ave size value (i.e. 160 nm for IMQ-LS) is based on scattering intensity and thus biased towards larger particles, and does not represent the true number-based size mean which may be significantly smaller, thus reducing the expected encapsulation efficiency. Nevertheless, it should be possible to improve the loading efficiency of the liposomes in the present work with further process optimization.

In earlier work, we had demonstrated that GLA intercalated into phospholipid-emulsified oil at the oil/water interface, causing a more negative zeta potential [38,43]. The same technique applied to anionic liposomes is not as discriminatory due to the relatively high negative charge of the liposomes themselves [38]; nevertheless, given the insolubility of GLA and its affinity for phospholipid structures [43,44], it is presumed that this TLR4 ligand localizes in the lipid bilayer in a similar manner to the other phospholipids. Moreover, in a previous report we employed *in vitro* bioactivity analysis and different order-of-mixing techniques to indicate that GLA formulated in anionic liposomes or oil-in-water emulsion is likely associated with the lipid particles rather than the bulk aqueous phase [45].

Ongoing work in our lab is seeking to enhance loading efficiency of liposomes containing IMQ by varying phospholipid concentration and liposome preparation techniques, such as replacing sonication with high pressure homogenization to achieve more reproducible and uniform particle size. However, even if loading efficiency remains somewhat low, the cost efficiency of IMQ-containing liposomes could still be quite favorable compared to other TLR7 ligands given that IMQ is available at approximately the same cost as phospholipid excipients (i.e. <$20/g) from generic manufacturers, which is not the case with newer imidazoquinoline-based TLR7 agonists.

### Th1 responses in vivo

**Figure 2:**
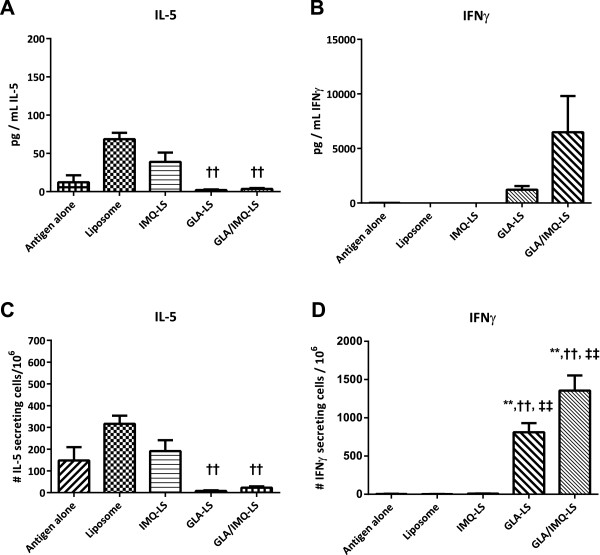
**Synergy adjuvant enhances Th1 responses.** Mice were immunized with either PbCSP antigen, the liposomal carrier, 20 μg IMQ in liposomes (IMQ-LS), 5 μg GLA in liposomes (GLA-LS) or the combination adjuvant (5 μg/20 μg, GLA/IMQ-LS), then spleen cells harvested and incubated with antigen. **Panels A and B**: ELISA determination of levels of secreted cytokines. **Panels C and D**: ELISPOT enumerations of the number of specific cytokine secreting cells. Significant differences between antigen alone, liposomes, IMQ liposomes, GLA liposomes, and IMQ/GLA liposomes were observed for the IFNγ ELISPOT (p-value < 0.0001), IL-5 ELISPOT (p-value = 0.0057), and the IL-5 ELISA (p-value = 0.0411). The differences for the IFNγ ELISA did not reach statistical significance. Symbols: * = significantly higher than Antigen alone;† = significantly higher than Liposomes alone; ‡ = significantly higher than IMQ-LS; § = significantly higher than GLA-LS. Single symbol: p < 0.05; Double symbol: p < 0.01; Shown are means with standard error.

Once well-characterized, liposomal adjuvants were available that contained GLA, IMQ, or both, their ability to mediate immune responses upon administration with the recombinant malaria antigen, PbCSP, was evaluated. The delivery system can potentially be used as an adjuvant for any appropriate vaccine, but we selected this malaria antigen as a model since we had had experience with adjuvanted formulations of this protein. While Th2 responses, as indicated by antigen-specific IL-5 secretion, were induced by immunization with empty or IMQ only-containing liposomes, these were reduced to essentially background levels with any GLA containing adjuvant (Figure 2). Thus, no difference could be seen in the ability to turn off IL-5 between the GLA alone and the GLA + IMQ adjuvant. In contrast, both GLA containing adjuvants increased Th1 responses, as indicated by antigen-specific IFNγ secretion. While the GLA alone liposomes resulted in robust secretion of IFNγ, the synergistic adjuvant was able to provide even higher IFNγ responses (Figure 2).

**Figure 3:**
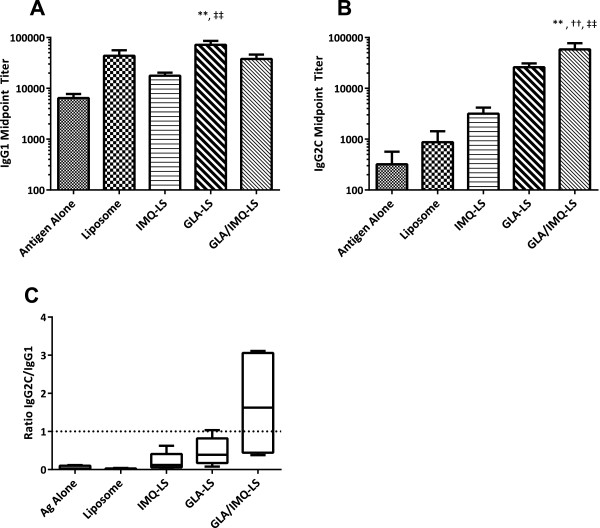
**Adaptive humoral responses induced by synergy adjuvants.** Sera were collected from mice after the second immunization and PbCSP antigen-specific IgG1 **(Panel A)** and IgG2c **(Panel B)** midpoint titers determined by ELISA. Consistent with the observed cytokine profile the combination induced higher levels of IgG2c, a marker for Th1 immunity in mice. **Panel C** – the ratio in titers highlights the ability of the combination to induce Th1 biased immunity. * = significantly higher than Antigen alone;† = significantly higher than Liposomes alone; ‡ = significantly higher than IMQ-LS; Single symbol: p < 0.05; Double symbol: p < 0.01. Shown are means with standard error.

To verify that the combination adjuvants resulted in a biologically active Th1 biased adaptive response, antibody responses were determined as a function of adjuvant (Figure 3). IgG1 in mice is reflective of a Th2 biased response and IgG2 is reflective of a Th1 bias. The trends seen with the antigen-specific spleen cell responses were reflected in the antibody responses: The adjuvants with TLR ligands gave more IgG2. The synergistic adjuvant gave the highest IgG2:IgG1 ratio consistent with the most Th1 biased immune response (Figure 3, Panel C).

## Conclusion

We report here the development of a synergistic adjuvant that is manufacturable and combines TLR4 and TLR7 ligands. We were able to develop a process by which anionic liposomes could be made that incorporate the TLR4 ligand GLA at the phospholipid bilayer interface and the TLR7 ligand IMQ in their interior. The liposome co-localizes the agonists allowing same cell activation of the external sensor TLR4 through GLA and TLR7 through imiquimod. Therefore, the same cell would experience simultaneous triggering of two innate sensors, resulting in an enhanced cytokine response.

## Methods

### Chemicals and reagents

Milled imiquimod (IMQ) was purchased from Chemagis (Bnei Brak, Israel); imiquimod was also obtained from Invivogen (San Diego, CA). Glucopyranosyl lipid adjuvant (GLA), 1,2-dipalmitoyl-*sn*-glycero-3-phosphocholine (DPPC), 1,2-dipalmitoyl-*sn*-glycero-3-phosphoglycerol (DPPG), 1,2-dioleoyl-*sn*-glycero-3-phosphocholine (DOPC), and 1,2,dipalmitoyl-3-trimethylammonium-propane (DPTAP) were purchased from Avanti Polar Lipids Inc. (Alabaster, AL). DOPC was also purchased from Lipoid (Newark, NJ). Cholesterol, and ammonium phosphate mono- and dibasic were purchased from J.T. Baker (San Francisco, CA). Lactic acid was purchased from Sigma-Aldrich (St. Louis, MO). Phosphate buffered saline (PBS) at pH 7.2 was purchased from Life Technologies (Grand Island, NY).

### Formulating liposomes

Liposome formulations were manufactured by first combining phospholipids, cholesterol, and optionally, depending on the desired type of adjuvant, GLA, in chloroform:methanol or chloroform:methanol:water, which was then evaporated overnight using a Genevac EZ-2 Plus Evaporator (Stone Ridge, New York). The dried components were rehydrated in either 100 mM lactic acid with or without 10 mg/ml IMQ, and then sonicated in a VWR 75D (West Chester, PA) or Crest Powersonic CP230D (Trenton, NJ) water batch sonicator at ~60°C for 1.5 – 3 hrs, until the formulation appeared homogeneous and translucent. This formulation was kept in a heated water bath prior to transferring 2.5 ml to a separate disposable PD-10 desalting column obtained from GE Healthcare Bio-Sciences AB (Uppsala, Sweden). The columns arrived pre-packed with Sephadex G-25 medium and were primed and subsequently eluted with 3.5 ml of PBS (pH 7.2). The buffer exchange step induces a ~30% dilution of each formulation and is employed to exchange the bulk aqueous phase external to the liposomes from lactic acid to PBS, and to remove non-encapsulated IMQ. GLA-LS formulations were then mixed with GLA-IMQ-LS formulations in order to generate final formulations with different doses of IMQ.

### Characterizing the formulations

Particle size of all liposomal formulations was monitored by dynamic light scattering (DLS) using the Malvern Instruments (Worcestershire, UK) Zetasizer Nano-S or Nano-ZS. Samples were prepared at 1:100 dilutions by combining 5 μl of each formulation with 500 μl of ultrapure water in a 1.5 ml polystyrene disposable cuvette. DLS measurements were then made three times on each cuvette. Formulations containing GLA were analyzed by reverse-phase high performance liquid chromatography (HPLC) with charged aerosol detection (CAD) to determine GLA concentration as previously published [38] except that in some cases the column employed was a Waters XBridge C18 (Milford, MA). Formulations containing IMQ were analyzed by UV–vis spectroscopy (Hitachi U-3900H, Tokyo, Japan) to confirm IMQ concentration via absorbance at 307 nm. The pH of the formulation before and after the buffer exchange was also measured. Liposome formulations containing IMQ were prepared in triplicates by combining 50 μl of sample with 950 μl of EtOH/HCl (98%/2%) into three separate disposable UV-cuvettes. IMQ concentration was extrapolated from a linear 5-point standard curve. pH was measured using a Mettler Toledo (Columbus, Ohio) MP225 pH meter and an Orion Ross semi-micro 8103BN pH probe obtained from Thermo Scientific (Waltham, MA). A 3-point calibration was performed prior to measurement with pH 4.00, 7.00, and 10.00 standard buffers. To determine whether IMQ was encapsulated in the liposomes, ultracentrifuged samples were prepared by transferring 200 μl of the liposome formulation into a 1.5 ml capacity ultracentrifuge tube and centrifuging at 160,000 × g at 4°C for three 3-hour cycles using an Optima™ MAX-XP Beckman-Coulter Ultracentrifuge (Indianapolis, IN). The supernatant was removed after each of the first two cycles and the pellet at the bottom of the tube was washed, each time with 1 ml of PBS with gentle mixing, followed by another 3 hours of ultracentrifugation at 160,000 × g at 4˚C, and subsequent removal of the supernatant. The liposome pellet was then lysed with 1.05 ml ethanol/concentrated hydrochloric acid (98%/2%), sonicated for ~5 mins, and diluted 1:20 in the same solvent mixture for spectrophotometric analysis.

### In-vitro stimulation using a whole blood assay (WBA)

After obtaining informed consent, heparinized whole blood was collected from healthy volunteers and 180 μl plated directly into 96-well round-bottom tissue culture plates. 20 μl of each formulation with the various innate stimuli were then added giving final well volumes of 200 μl. Each stimulation condition was conducted in duplicate. IMQ from Invivogen was suspended in dH_2_O to a concentration of 5 mg/ml. GLA-AF was manufactured as described in Orr *et al.*[38] at a concentration of 1 mg/ml. IMQ and GLA were diluted in PBS prior to addition, either separately and in combination, to blood, which was then incubated at 37°C, 5% CO_2_ for 24 h. After incubation, 100 μl of the plasma supernatant was carefully extracted and cytokine content measured by ELISA: Mip-1β (R & D Systems, Minneapolis, MN); IL-8, IL-12p70, and IFNγ (eBioscience, San Diego, CA).

### Mice and immunizations

*Plasmodium berghei* circumsporozoite protein (PbCSP) was expressed and purified from *E. coli* using the codon-harmonized construct kindly provided by Dr. Evelina Angov from the Walter Reed Army Institute of Research. All animal protocols were approved by the IDRI institutional animal care and use committee. Female C57BL/6 mice were purchased from Charles River Laboratories (Wilmington, MA) and maintained in specific pathogen-free conditions. Mice, 6–8 weeks of age, were immunized intramuscularly three times in two-week intervals by injection at the base of the tail. For immunization, recombinant protein was formulated with adjuvant to provide a total of 2 μg protein/injection with various doses of the adjuvant in a total volume of 0.1 ml. The adjuvant doses corresponded to 20 μg IMQ and 5 μg GLA for both the single and combined adjuvants.

### Antibody analyses

Blood was collected from the retro-orbital sinus two weeks after the second immunization and sera prepared. Sera were stored at 4°C until antigen-specific antibody responses were analyzed by ELISA. Briefly, ELISA plates (Nunc, Rochester, NY) were coated with 1 μg/ml antigen in 0.1 M bicarbonate buffer and blocked with 0.1% BSA-PBS. Following washes in PBS/Tween, serially diluted serum samples were added. After incubation and further washes, either anti-mouse IgG-HRP, anti-mouse-IgG2c-HRP or anti-mouse IgG1-HRP were added (all Southern Biotech, Birmingham, AL). After incubation and washing, ABTS-H_2_O_2_ (Kirkegaard and Perry Laboratories, Gaithersburg, MD) was added to the plates to reveal any reactions, which were stopped by the addition of 0.1 N H_2_SO_4_. Plates were analyzed at 405 nm (EL_X_808, Bio-Tek Instruments Inc, Winooski, VT). Midpoint titers were determined as EC50 values from weighted curve fits using the GraphPad Prism package V 6.03.

### Cell preparations and antigen stimulation assays

Six weeks after the final immunization, spleens were removed and single cell suspensions prepared. Mononuclear cells were enumerated using a ViaCount assay with a PCA system (Guava Technologies, Hayward, CA). To determine overall cytokine production, spleen cells were cultured at 2 × 10^5^ cells per well in duplicate in a 96-well plate (Corning Incorporated, Corning, NY) in RPMI-1640 supplemented with 10% heat-inactivated FCS and 50,000 Units penicillin/streptomycin (Invitrogen), in the presence of 10 μg/ml protein. Culture supernatants were harvested after 4 days and IFNγ/IL-5 content determined by ELISA, according to the manufacturer’s instructions (eBioscience, San Diego, CA).

To determine the number of cells producing each cytokine, multiScreen 96-well filtration plates (Millipore) were coated with rat anti-mouse IL-5 or rat anti-mouse IFNγ capture antibody (both eBioscience) and incubated overnight at 4˚C. Plates were washed with PBS, blocked with RPMI 1640 and 10% FBS for at least 1 h at room temperature, and washed again. Spleen cells were then added at 2 × 10^5^ cells/well and stimulated with media or antigen (10 μg/ml) for 48 h at 37˚C. The plates were then washed with 0.1% PBS–Tween 20 and incubated overnight with a biotin-conjugated rat anti-mouse IL-5 or IFNγ secondary antibody (eBioscience) diluted in 0.1% PBS–Tween 20/0.5% BSA. The filters were developed using the VectaStain ABC avidin peroxidase conjugate and Vectastain AEC substrate kits (Vector Laboratories, Burlingame, CA) according to the manufacturer’s protocol. The reaction was stopped by washing the plates with deionized water. Plates were dried in the dark, and spots were counted on an automated ELISPOT reader (C.T.L. Series 3A Analyzer; Cellular Technology Ltd., Shaker Heights, OH) and analyzed with ImmunoSpot software (Cellular Technology Ltd).

### Statistical methods

Five mice were immunized for each treatment with two subsamples taken per mouse. These subsamples were handled by treating mice within each treatment as a nested random effect to allow the separation of the variability between mice from the variability within each mouse. In order to test Th1 and Th2 responses in vivo using ELISA determination of levels of secreted cytokines and ELISPOT enumerations of the number of specific cytokine screening cells for both IL-5 and IFNγ secretions, four one-way ANOVA tests were used. Each test was used to compare mean differences between mice immunized with either antigen, the liposomal carrier, IMQ in liposomes, GLA in liposomes, or the combination adjuvant. Tukey’s procedure was used to test pairwise comparisons between treatments if significant treatment effects were found. Consideration of the variance estimates for the nested factor indicated that, in all cases, the majority of the variation in the data was due to variation between mice rather than variation among the subsamples taken from individual mice. All hypothesis testing was done at the 95% level.

## Abbreviations

MPL: 3-O-desacyl-4′-monophosphoryl lipid A; IFNγ: Interferon gamma; IL: Interleukin; MIP-1β: Macrophage inflammatory protein 1-beta; TLR: Toll-Like Receptor; GLA: Glucopyranosyl lipid adjuvant; IMQ: Imiquimod.

## Competing interests

The authors declare that they have no competing interests.

## Authors’ contributions

CF and SS performed the manufacturing and characterization studies. MD and JV performed the *in vivo* immunology; JG performed the cell-based assays. EM and DCo performed statistical analyses. DC and SR conceived of the study, and participated in its design and coordination and helped to draft the manuscript. All authors read and approved the final manuscript.
